# Efficacy and safety of oral upadacitinib-topical ruxolitinib combination therapy in the treatment of progressive nonsegmental vitiligo

**DOI:** 10.1016/j.jdin.2025.04.014

**Published:** 2025-06-12

**Authors:** Weiran Li, Jinping Gao, He Huang, Tingting Zhu, Xianfa Tang, Ruochen Zhang, Xiaojian Chen, Yang Wang, Yaohua Zhang, Xuejun Zhang, Yong Cui, Youwen Zhou, Bo Zhang

**Affiliations:** a Department of Dermatology, The First Affiliated Hospital of Anhui Medical University, Hefei, Anhui, China; b Department of Dermatology, The Fourth Affiliated Hospital of Soochow University (Suzhou Dushu Lake Hospital), Medical Center of Soochow University, Suzhou, Jiangsu, China; c Dermatology Center in Bo’ao Super Hospital, Qionghai, Hainan, China; d Department of Dermatology, Institute of Dermatology, Huashan Hospital, Fudan University, Shanghai, China; e Department of Dermatology, China-Japan Friendship Hospital, Beijing, China; f Department of Dermatology and Skin Science, University of British Columbia, Vancouver, Canada

**Keywords:** combination therapy, efficacy, JAK inhibitor, nonsegmental vitiligo, progressive, ruxolitinib, upadacitinib

*To the Editor:* Vitiligo is an immune-mediated depigmenting disease, with the most common form being non-segmental vitiligo (NSV).^1^ As studies found that interferon gamma/Janus kinase (JAK)/signal transducer and activator of transcription pathway played crucial role in the pathogenesis of vitiligo, JAK inhibitors could potentially be a promising therapeutic approach.^2^ Ruxolitinib cream, which selectively inhibits JAK1/JAK2, has been approved for the treatment of vitiligo.^3^ Upadacitinib, an oral selective JAK1 inhibitor, has also demonstrated effective repigmentation in patients with NSV.^4^^,^^5^

In this study, oral upadacitinib-1.5% ruxolitinib cream combination was evaluated for efficacy and safety in treating 30 progressive NSV patients (Table I). Upadacitinib was given at a dose of 15 mg once daily and 1.5% ruxolitinib cream was applied twice daily for 24 weeks. Patients were assessed at baseline, week 12, and week 24 for total and area-specific Vitiligo Area Severity Index (VASI) scores, adverse events (AEs), as well as liver/kidney function tests and blood lipid profiles.

**Table Ⅰ:** Demographic and clinical characteristics of vitiligo patients

Patient no.	Sex	Age, y	Body site involvement	Disease duration, mo(s)	Family history	Koebner phenomenon
1	Female	13	Face, hand, foot, extremities	60	N	N
2	Male	27	Face, neck, hand, foot, trunk, extremities	93	N	Y
3	Male	36	Face, hand, foot, trunk, extremities	240	N	N
4	Female	42	Neck, hand, foot, trunk, extremities	108	N	N
5	Male	60	Face, neck, hand, foot, trunk, extremities	24	N	N
6	Male	54	Hand, trunk	84	N	N
7	Male	45	Face, hand, foot, trunk	240	N	N
8	Male	36	Face, hand, extremities	144	N	N
9	Male	40	Face, neck, hand, trunk, extremities	60	N	N
10	Female	16	Face, neck, hand, trunk, extremities	48	N	N
11	Female	49	Face, neck, hand, trunk, extremities	24	N	N
12	Male	40	Face, trunk, extremities	18	N	N
13	Female	49	Face, neck, hand, trunk	24	N	N
14	Female	55	Neck, foot, trunk	10	N	N
15	Male	48	Face, hand, trunk, extremities	456	Y	Y
16	Male	36	Face, hand, trunk, extremities	156	N	N
17	Female	13	Face, hand	48	Y	N
18	Female	49	Face, neck, hand, foot, trunk, extremities	120	N	N
19	Female	49	Face, hand	2	N	N
20	Male	30	Face, hand, trunk, extremities	72	N	N
21	Female	55	Face, neck, trunk, extremities	12	N	N
22	Male	35	Face, neck, hand, trunk, extremities	168	N	Y
23	Female	39	Face, neck, hand	36	N	N
24	Female	28	Face, trunk, extremities	24	N	N
25	Male	41	Face, neck, hand, foot, trunk, extremities	72	N	N
26	Male	46	Face, hand, foot, trunk, extremities	72	N	N
27	Male	15	Face, hand, foot, trunk, extremities	1	N	Y
28	Female	55	Face, hand, foot, trunk, extremities	600	N	N
29	Male	15	Face, neck, hand, foot	12	N	Y
30	Male	60	Face, neck, hand, foot, trunk, extremities	24	N	N
Age, y (range)				13-60		
Average age, y, median (range)				40.5 (29.5-45)		
BSA,%, median (range)				2.6 (1.15-6.65)		
T-VASI, median (range)				2.35 (1-6.65)		
F-VASI, median (range)				0.2 (0.1-0.5)		

Data presented as median (range) due to non-normal distribution.

*BSA*, Body surface area; *F-VASI*, Facial Vitiligo Area Scoring Index; *T-VASI*, Total Vitiligo Area Scoring Index.

The total VASI at the baseline was 2.35 (1-6.65) and dropped to 1.57 (0.57-6.33) at week 12 (*P* < .0001) and 1.27 (0.46-6.12) at week 24 (*P* < .0001), reflecting an average improvement of 23.19% (rang 2% to 79.84%) and 30.99% (range 3.92% to 97.58%), respectively. One patient achieved Total Vitiligo Area Scoring Index (T-VASI)90, 2 patients achieved T-VASI75 and 6 patients achieved T-VASI50 at week 24. Among 27 patients with facial vitiligo, the facial VASI at the baseline was 0.2 (0.1-0.5) and dropped to 0.135 (0.05-0.27) at week 12 (*P* < .0001) and 0.1 (0.01-0.20) at week 24 (*P* < .0001), reflecting an average improvement of 36.66% (range 0% to 100%) and 56.17% (range 0% to 100%), respectively. Five patients achieved F-VASI90, and 8 patients achieved Facial Vitiligo Area Scoring Index (F-VASI)50 at week 12, then 8 patients achieved F-VASI90, 10 patients achieved F-VASI75 and 15 patients achieved F-VASI50 at week 24. Significant improvements were observed in both the distal extremities (hand/foot) VASI scores of 28 patients (*P* = .0033) and the trunk VASI scores of 24 patients (*P* < .0001) at week 24. Twenty patients responded on the extremities with significant improvement at 12 week (*P* = .0009) as well as 24 week (*P* = .0005). (Table II) (Supplementary Fig 1, available via Mendeley at https://data.mendeley.com/datasets/5gp4xy63xv/1). AEs were reported by 4 patients (13.33%), 3 had experienced acne, one had elevated high-density lipoprotein cholesterol. No severe AEs occurred.

**Table II:** Treatment outcomes of patients

Patient no.	BSA	T-VASI			F-VASI			D-VASI			Extremities-VASI			Trunk-VASI		
	W0/W12/W24	W0/W12/W24	Response (W12, %)	Response (W24, %)	W0/W12/W24	Response (W12, %)	Response (W24, %)	W0/W12/W24	Response (W12, %)	Response (W24, %)	W0/W12/W24	Response (W12, %)	Response (W24, %)	W0/W12/W24	Response (W12, %)	Response (W24, %)
1	2.5/2.5/2.5	2.5/2.45/2.095	2.00	16.20	0.5/0.45/0.375	10.00	25.00	0.8/0.8/0.8	0.00	0.00	0.8/0.8/0.56	0.00	30.00	0.4/0.4/0.36	0.00	10.00
2	11.2/10.9/10.3	9.5/8.5/7.145	10.53	24.79	0.5/0.15/0.05	70.00	90.00	2.025/2.025/1.845	0.00	8.89	3.975/3.975/2.95	0.00	25.79	2.7/2.25/2.25	16.67	16.67
3	26.4/25.4/25.2	26.4/24.425/21.335	7.48	19.19	1.4/0.1/0.05	92.86	96.43	3.4/3.4/3.06	0.00	10.00	13.5/12.825/10.125	5.00	25.00	8.1/8.1/8.1	0.00	0.00
4	2.07/2.04/0.12	2.07/0.5725/0.05	72.34	97.58	0/0/0	/	/	0.07/0.035/0.0075	50.00	89.29	0.15/0.0375/0.02	75.00	86.67	1.8/0.45/0.0125	75.00	99.31
5	10/8.4/9.5	9.43/8.07/8.045	14.42	14.69	0.45/0.36/0.27	20.00	40.00	2.52/2.51/2.225	0.40	11.71	3.76/2.5/3.55	33.51	5.59	1.8/1.8/1.8	0.00	0.00
6	0.85/0.7/0.6	0.85/0.6/0.475	29.41	32.35	0/0/0	/	/	0.5/0.5/0.45	0.00	10.00	0/0/0	/	/	0.35/0.05/0.025	85.71	92.86
7	1.45/0.8/0.79	1.45/0.8/0.79	44.83	45.52	0.05/0.04/0.03	20.00	40.00	1.2/0.73/1.2	39.17	0.00	0/0/0	/	/	0.2/0.03/0.2	85.00	0.00
8	6.1/5.9/5.6	6.1/5.6/4.95	8.20	18.85	0.3/0/0	100.00	100.00	0.8/0.8/0.45	0.00	43.75	5/4.8/4.5	4.00	10.00	0/0/0	/	/
9	0.5/0.25/0.36	0.5/0.25/0.36	50.00	28.00	0.1/0.05/0.01	50.00	90.00	0.2/0.2/0.35	0.00	−75.00	0/0/0	/	/	0.2/0/0	100.00	100.00
10	2.2/0.4/0.3	1.91/0.385/0.25	79.84	86.91	0.18/0.15/0.15	16.67	16.67	0.15/0.1/0.05	33.33	66.67	0.81/0.135/0.05	83.33	93.83	0.72/0/0	100.00	100.00
11	0.7/0.4/0.3	0.3625/0.2/0.125	44.83	65.52	0.0375/0.025/0.0125	33.33	66.67	0.025/0.025/0.0125	0.00	50.00	0/0/0	/	/	0/0/0	/	/
12	4.85/4/3.9	4.85/3.73/2.55	23.09	47.42	1.3/1.3/1.2	0.00	7.69	0/0/0	/	/	0.85/0/0	100.00	100.00	2.7/2.43/1.35	10.00	50.00
13	2.7/2.7/1.95	2.2/1.695/1.155	22.95	47.50	0.5/0.27/0.18	46.00	64.00	0.1/0.025/0	75.00	100.00	0/0.2/0.135	/	/	1.5/1/0.75	33.33	50.00
14	1.4/1.12/0.71	1.05/0.635/0.38	39.52	63.81	0/0/0	/	/	0.225/0.225/0.075	0.00	66.67	0/0/0	/	/	0.6/0.4/0.3	33.33	50.00
15	6.6/6.5/6.3	6.6/6.5/6.3	1.52	4.55	0.3/0.2/0.1	33.33	66.67	1/1/1	0.00	0.00	0.8/0.8/0.7	0.00	12.50	4.5/4.5/4.5	0.00	0.00
16	3.5/2/1.5	3.5/1.16/0.98	66.86	72.00	2.5/0.25/0.125	90.00	95.00	0.1/0.1/0.09	0.00	10.00	0.3/0.27/0.225	10.00	25.00	0.6/0.54/0.54	10.00	10.00
17	0.6/0.5/0.5	0.6/0.5/0.5	16.67	16.67	0.2/0.2/0.2	0.00	0.00	0.4/0.3/0.3	25.00	25.00	0/0/0	/	/	0/0/0	/	/
18	4/3.6/3.41	3.235/2.935/2.71	9.27	16.23	0.06/0.06/0.01	0.00	83.33	1.275/0.975/0.8	23.53	37.25	1.6/1.6/1.6	0.00	0.00	0.2/0.2/0.2	0.00	0.00
19	0.4/0.35/0.35	0.4/0.3125/0.275	21.88	31.25	0.2/0.2/0.2	0.00	0.00	0.2/0.1125/0.075	43.75	62.50	0/0/0	/	/	0/0/0	/	/
20	0.61/0.61/0.59	0.575/0.56/0.4895	2.61	14.87	0.15/0.135/0.117	10.00	22.00	0.01/0.01/0.01	0.00	0.00	0.1/0.1/0.1	0.00	0.00	0.315/0.315/0.2625	0.00	16.67
21	6.3/6.3/6.2	6.3/5.9/5.9	6.35	6.35	0.5/0.375/0.225	25.00	55.00	0/1/0.1	0.00	0.00	1/0.2/1.2	80.00	−20.00	4.3/4.3/4.3	0.00	0.00
22	53/51.95/49.9	52/49.5125/46.9	4.78	9.81	2/0.9/0.4	55.00	80.00	2/2/2	0.00	0.00	12/10.8/9	10.00	25.00	33/33/33	0.00	0.00
23	0.22/0.17/0.17	0.16/0.1225/0.0975	23.44	39.06	0.1125/0.075/0.05	33.33	55.56	0.01/0.01/0.01	0.00	0.00	0/0/0	/	/	0/0/0	/	/
24	1.5/1.45/1.45	1.2125/1.175/1.165	3.09	3.92	0.1/0.1/0.09	0.00	10.00	0/0/0	/	/	0.325/0.325/0.325	0.00	0.00	0.7875/0.75/0.75	4.76	4.76
25	2.9/2.35/2.35	2.825/2.06/2.06	27.08	27.08	0.225/0.135/0.135	40.00	40.00	2/1.45/1.45	27.50	27.50	0/0/0	/	/	0.1/0.1/0.1	0.00	0.00
26	8.81/8.8/8.8	7.809/6.75/6.675	13.56	14.52	0.009/0.009/0	0.00	100.00	4.5/4.45/4.375	1.11	2.78	1.8/1.3/1.3	27.78	27.78	1.5/1.5/1	0.00	33.33
27	2.23/1.72/1.72	1.55/1.445/1.37	6.77	11.61	0.005/0/0	100.00	100.00	0.1/0.6/0.525	−500.00	−425.00	1.42/0.825/0.825	41.90	41.90	0.025/0.02/0.02	20.00	20.00
28	6.8/6.71/6.51	6.8/6.269/6.0575	7.81	10.92	0.1/0.009/0.0075	91.00	92.50	3/2.9/2.9	3.33	3.33	2.2/2.01/1.845	8.64	16.14	1.5/1.35/1.305	10.00	13.00
29	1.25/1/1	1.25/1/0.9	20.00	28.00	0.15/0.1/0.09	33.33	40.00	1/0.9/0.81	10.00	19.00	0/0/0	/	/	0/0/0	/	/
30	10/8.4/9.5	9.43/8.07/8.045	14.42	14.69	0.45/0.36/0.27	20.00	40.00	2.52/2.51/2.225	0.40	11.71	3.76/2.5/3.55	33.51	5.59	1.8/1.8/1.8	0.00	0.00

*BSA*, Body surface area; *D-VASI*, distal extremities (hand/foot) Vitiligo Area Scoring Index; *E-VASI*, Extremities Vitiligo Area Scoring Index; *F-VASI*, Facial Vitiligo Area Scoring Index; *T-VASI*, Total Vitiligo Area Scoring Index; *Trunk-VASI*, Trunk Vitiligo Area Scoring Index.

In summary, we combined 2 kinds of JAK inhibitions in treating progressive NSV patients for 24 weeks. All participants demonstrated an effective treatment response, showing repigmentation without disease progression, indicating that upadacitinib has a potent ability to halt the progression of vitiligo. The rates of T-VASI50 (20%) and F-VASI75 (33%) in our study were greater than the phase 2 clinical trials of upadacitinib (T-VASI50 11.6%, F-VASI75 19.1%)^2^ and the phase 3 trials of ruxolitinib cream (F-VASI75: TRuE-V1 29.8% and TRuE-V2 30.9%).^3^ It demonstrated notable improvement of T-VASI and F-VASI in progressive NSV patients treated with upadacitinib and ruxolitinib cream. The combination therapy might also be effective on trunk and extremities, including hand and foot. Further studies with larger sample sizes are needed to determine the efficacy and safety of this combination treatment for vitiligo.

## Conflicts of interest

None disclosed.
